# Assessment of major centelloside ratios in *Centella asiatica* accessions grown under identical ecological conditions, bioconversion clues and identification of elite lines

**DOI:** 10.1038/s41598-022-12077-9

**Published:** 2022-05-17

**Authors:** Renju Kunjumon, Anil John Johnson, Rajani Kurup Sukumaryamma Remadevi, Sabulal Baby

**Affiliations:** 1 Phytochemistry and Phytopharmacology Division, Jawaharlal Nehru Tropical Botanic Garden and Research Institute, Pacha-Palode, Thiruvananthapuram, Kerala 695562 India; 2 University of Kerala, Thiruvananthapuram, Kerala 695034 India

**Keywords:** Natural variation in plants, Plant ecology, Secondary metabolism

## Abstract

Centellosides viz*.*, asiatic acid, madecassic acid, asiaticoside, madecassoside, are the major bioactive molecules in *Centella asiatica*. In this study madecassic acid:asiatic acid, madecassoside:asiaticoside (C6-hydroxylation *versus* non-hydroxylation) and asiaticoside:asiatic acid, madecassoside:madecassic acid (C28-glycoside *versus* aglycone) ratios in 50 *C. asiatica* accessions originally collected from their natural habitats in south India and grown under identical ecological conditions for six generations were determined using validated HPTLC-densitometry protocols. Asiatic acid, madecassic acid, asiaticoside and madecassoside contents ranged from 0.00–0.29% (average 0.03 ± 0.06%; 28 accessions recorded asiatic acid content as zero), 0.02–0.72% (0.12 ± 0.13%), 0.04–2.41% (0.44 ± 0.52%) and 0.15–5.27% (1.59 ± 1.26%), respectively. Distinctly, C6-hydroxylated (madecassic acid:asiatic acid 4.00, madecassoside:asiaticoside 3.61) and C28-glycosylated (asiaticoside:asiatic acid 14.67, madecassoside: madecassic acid 13.25) centellosides dominated over the respective non-derivatized entities. Our results infer that both C6-hydroxylation by CYP450-dependent monooxygenases and C28-glycosylation by UDP-Glc glucosyltransferases are dominant bioconversion steps in *C. asiatica*. Besides, this study discovered six elite lines of *C. asiatica*, with their (asiaticoside + madecassoside) contents above the industrial benchmark (≥ 4%) from south India. Two elite clones with asiaticoside contents ≥ 2% were also identified. Standardization of the agrotechniques of these elite lines could lead to their industrial applications. Further, this study emphasizes the need for standardizing all four centellosides as biomarkers in *C. asiatica* raw drugs, pharmaceutical and cosmetic products.

## Introduction

**Scheme 1:**
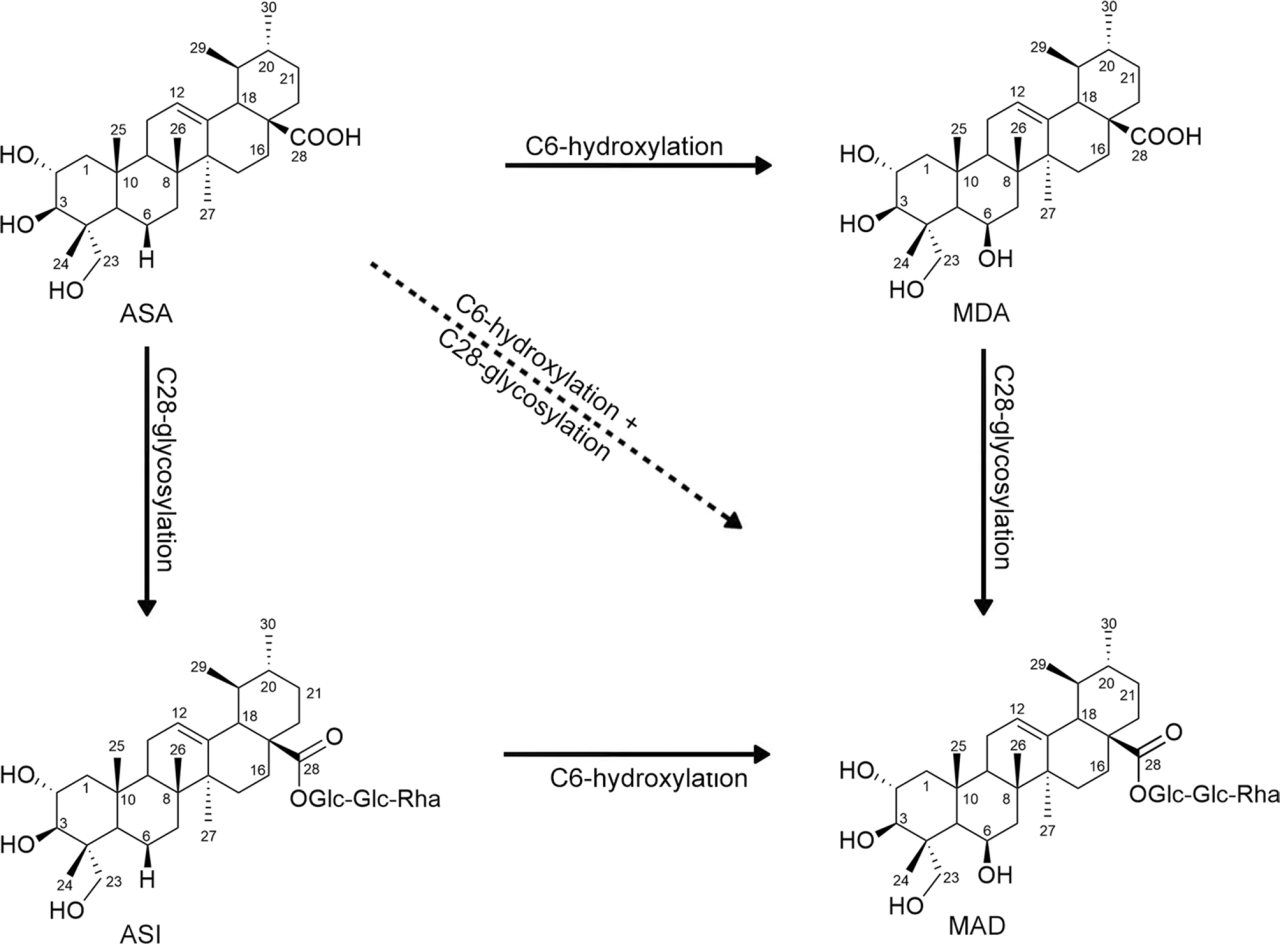
Four major centellosides in *C. asiatica*, asiatic acid (ASA), madecassic acid (MDA), asiaticoside (ASI) and madecassoside (MAD), and their bioconversions through C6-hydroxylation and C28-glycosylation reactions.

*Centella asiatica* (L.) Urban (CA, family Apiaceae) is a pharmaceutically important medicinal herb, known for its neuroprotective, memory enhancing, cardioprotective, antioxidant, anti-inflammatory, anticancer and wound healing activities^1,2^. India and Madagascar are the two major geographical sources of CA. So far, over 130 secondary metabolites were isolated from CA^1^. The prominent CA metabolites are the four centellosides (sapogenins: asiatic acid (ASA), madecassic acid (MDA); saponins: asiaticoside (ASI), madecassoside (MAD)) (Scheme 1). Centellosides are triterpene (6 isoprene units) saponins/sapogenins bearing an ursane or oleanane skeleton; CA centellosides viz*.*, ASA, MDA, ASI, MAD, are ursane-type triterpenes/glycosides. Of these MDA and MAD have hydroxyl (–OH) substitutions (hydroxylation) at C6 instead of the (–H) in ASA and ASI (Scheme 1). In both ASA and MDA, C28s are linked to –OH groups and the modifications in their saponins (ASI, MAD) are three sugar units (-Glu-Glu-Rha), and they are formed by glycosylation (at the C28s)^3^. The molecular formulae and mol. weights of CA aglycones and glycosides are ASA C_30_H_48_O_5_, 488.70 Da, MDA C_30_H_48_O_6_, 504.71 Da, ASI C_48_H_78_O_19_, 959.12 Da and MAD C_48_H_78_O_20_, 975.12 Da.

Studies on the biosynthesis of centellosides are gradually evolving^3–12^. The triterpenoid saponin skeletons in plants, which include oleanane, ursane, lupane and dammarane types, are synthesized via the isoprenoid pathway through farnesyl diphosphate (FPP) followed by cyclization of 2,3-oxidosqualene by 2,3-oxidosqualene cyclase (OSC). In CA, the genes involved in the main pathway to triterpenoid formation include farnesyl diphosphate synthase (*CaFPS*), squalene synthase (*CaSQS*), oxidosqualene synthase (*CaOSQs*) and the putative β-amyrin synthase (*CabAs*), an oxidosqualene cyclase, subsequently identified as a dammarenediol synthase (*CaDDs*)^4,8,9^. Modifications to the triterpenoid backbone include oxidation, substitution or glycosylation by enzymes such as CYP450-dependent monooxygenases and UDP-Glc glucosyltransferases (UGTs). The aglycones (sapogenins) undergo oxidation at various positions in the C30 triterpenoid skeleton through CYP450 monooxygenase enzymes^13,14^. CYP716A53v2 is reported to hydroxylate C6 of the dammarane-type tetracyclic sapogenin, protopanaxadiol, in *Panax ginseng* to form protopanaxatriol^15^. A recent study specifically identified CYP716 enzymes effecting the bioconversion of ursane and oleanane pentacyclic triterpenoid skeletons to their 6β-hydroxy derivatives (C6-hydroxylation) in CA^13,16^. CYP450s and their functional roles in plants are least explored so far. UGTs are pivotal enzymes in the process of glycosylation in plants, contributing to the biosynthesis of medicinally important secondary metabolites. In saponin biosynthesis, UGTs catalyze the transfer of UDP linked sugar moieties to the triterpenoid skeleton^4,9,17^. Glycosides, ASI and MAD, in CA are formed by glycosylation of ASA and MDA, respectively, catalyzed by UGTs which link Glu-Glu-Rha to the C28 carboxyl groups^3,4,9,12^. Hydroxylation and glycosylation change the physicochemical properties and enhance the biological potentials of triterpenoids^12,18,19^. In ASI and MAD, the triterpenoid structures (aglycone) are hydrophobic and are linked to hydrophilic sugar chains (glycone)^12,18–20^. The surface-active properties of saponins (*sapo* (Latin) = soap; soap-like surfactants that form long-lasting bubbles on shaking an aqueous solution) are distinguishing factors of these amphiphilic compounds from other glycosides. This is of considerable significance in drug design, and in CA, the glycosylated entities (saponins: ASI, MAD) are the prime target molecules in neuroprotection, memory enhancing, wound healing and skin protection.

Pentacyclic triterpenes (centellosides) are accumulated in CA in their glycoside (ASI, MAD) forms rather than as aglycones (ASA, MAD); and the glycoside to aglycone ratios influence the efficacy of CA extracts and its pharmaceutical and skin care products^21–23^. Several studies quantified the four major centellosides (ASA, MDA, ASI, MAD) by various analytical techniques^20,21,24–26^, whereas a few reports estimated only one or two (not all four) of these terpenoids in CA (examples, Devkota et al.^27^; Thomas et al.^28^; Prasad et al.^29^). But, most of these quantification studies are on limited number of samples from various genetic/ecological origins. Genetic and environmental parameters significantly affect the production of secondary metabolites in plants, viz*.*, centellosides in CA^20,26,28,30–32^. Therefore, the C6-hydroxylation (MDA, MAD) *versus* non-hydroxylation (ASA, ASI) and C28-glycoside (ASI, MAD) *versus* aglycone (ASA, MDA) ratios (Scheme 1) in CA are determined by the variations in the genes (and enzymes) involved in their biosynthesis, and to a lesser extent by the ecological parameters^4,5,20^. Otherwise, genetically these ratios depend on the presence and activity of enzymes involved in C6-hydroxylation and C28-glycosylation^20^. In CA cell cultures, high production of centellosides is achieved by growth regulators and elicitors (examples, methyl jasmonate, salicylic acid), and they presumably modulate the expression of certain genes involved in their biosynthesis^10^. CA cell cultures are also capable of converting precursors like α-amyrin into centellosides with very high efficiency^6^. Plant cell cultures are able to carry out regio- and stereoselective hydroxylation, hydrogenation and glycosylation of exogenous substrates, and biotransforming them into other compounds with improved pharmacological actions^10^. Therefore, the (C6-hydroxylation *versus* non-hydroxylation) and (C28-glycoside *versus* aglycone) ratios in CA cell cultures are influenced by the biotransformations induced by their growth conditions.

Here, we explore the ratios of the four major centellosides viz*.*, ASA, MDA, ASI, MAD, formed by C6-hydroxylation and/or C28-glycosylation of their precursor (ASA) (Scheme 1), in 50 accessions of CA originally collected from their natural habitats in south India and grown under identical ecological conditions for six generations. The study is conceived to derive bioconversion clues on the four centellosides by nullifying their ecological variations. We also address the significance of using the four centellosides as biomarkers in CA extracts and products. Moreover, these 50 CA accessions under study are scrutinized for elite lines based on industrial benchmarks of the contents of centellosides.

## Materials and methods

### Chemicals and reagents

ASI (≥ 98.5% HPLC), MAD (≥ 95% HPLC) and ASA (≥ 98% HPLC) were purchased from Sigma Aldrich (St. Louis, Missouri, US). MDA (≥ 96%) was procured from Santa Cruz Biotechnology (Santa Cruz, Dallas, USA). Silica gel TLC plates (60 F_254,_ 20 × 10 cm, 0.2 mm thickness) were obtained from E. Merck, Germany. Solvents used for the HPTLC analysis were of analytical or HPLC grade.

### Collection of plant materials, growing conditions

Fifty CA accessions were collected from their natural habitats in various agro-climatic regions of the south Indian states of Kerala, Tamil Nadu and Karnataka (Table 1), taxonomically authenticated by Dr. Mathew Dan, Principal Scientist of Jawaharlal Nehru Tropical Botanic Garden and Research Institute (JNTBGRI), Thiruvananthapuram and a voucher specimen (91008) was preserved at JNTBGRI Herbarium (TBGT) for future reference. These CA accessions were planted in the Field Gene Bank (FGB) of JNTBGRI in an evenly spread potting medium (1:1 top soil-sand) on level ground with uniform spacing of 30 cm apart in a randomized block design, without any external input of organic manure or chemical fertilizer, watered as and when needed, and maintained under uniform environmental conditions for a minimum of 3 years. For phytochemical analysis, aerial parts of four replications of six generation vegetatively propagated plants in flowering stage were collected in May 2019, dried in an oven at 40 °C and powdered. CA collections in this work were made as part of one of the Programme Support projects hosted by JNTBGRI and funded by Department of Biotechnology (DBT), Government of India; and collection of these plant materials is in compliance with relevant institutional, national, and international guidelines and legislation.

**Table 1:** Quantitative analyses of ASA, MDA, ASI and MAD in fifty accessions of CA, originally collected from various agro-climatic regions of south India and vegetatively propagated and maintained in a Field Gene Bank under identical environmental conditions for six generations.

Accession no.	Collection location	State	Altitude (m)	ASA (%)	MDA (%)	ASI (%)	MAD (%)
1/Ca 07	Perumathura, Thiruvananthapuram	Kerala	150	ND	0.37 ± 0.02	0.11 ± 0.01	0.40 ± 0.01
2/Ca 08	Madanvila, Thiruvananthapuram	Kerala	240	ND	0.30 ± 0.03	0.36 ± 0.01	1.02 ± 0.01
3/Ca 09	Anakunnam, Pallikal, Thiruvananthapuram	Kerala	120	ND	0.34 ± 0.03	0.25 ± 0.01	0.83 ± 0.01
4/Ca 10	Pallikal, Thiruvananthapuram	Kerala	120	ND	0.05 ± 0.01	0.40 ± 0.01	2.32 ± 0.03
5/Ca 11	Parippally, Kollam	Kerala	90	0.03 ± 0.01	0.41 ± 0.02	0.49 ± 0.01	1.63 ± 0.02
6/Ca 12	Kazhuthurutty, Aryankavu, Kollam	Kerala	200	0.05 ± 0.00	0.14 ± 0.01	0.65 ± 0.02	0.84 ± 0.04
7/Ca 13	Kaithakkadu, Kulathupuzha, Kollam	Kerala	170	0.01 ± 0.00	0.03 ± 0.00	0.13 ± 0.01	0.79 ± 0.01
8/Ca 15	Kulathupuzha, Kollam	Kerala	170	0.01 ± 0.02	0.39 ± 0.00	0.25 ± 0.01	1.03 ± 0.02
9/Ca 17	Aryankavu, Kollam	Kerala	284	0.08 ± 0.00	0.05 ± 0.00	0.58 ± 0.03	1.57 ± 0.05
10/Ca 18	Arippa, Kollam	Kerala	197	ND	0.06 ± 0.01	0.14 ± 0.01	1.17 ± 0.04
11/Ca 19	Nedungolam, Kollam	Kerala	16	0.18 ± 0.01	0.05 ± 0.00	0.29 ± 0.03	2.02 ± 0.05
12/Ca 21	Pandalam, Pathanamthitta	Kerala	100	0.02 ± 0.01	0.05 ± 0.00	0.67 ± 0.03	1.57 ± 0.05
13/Ca 22	Ranni, Pathanamthitta	Kerala	160	0.01 ± 0.00	0.04 ± 0.01	0.55 ± 0.02	2.97 ± 0.05
14/Ca 23	Koodal, Pathanamthitta	Kerala	140	ND	0.05 ± 0.00	0.11 ± 0.01	0.48 ± 0.02
15/Ca 25	KGMOA House, Pathanamthitta	Kerala	60	ND	0.03 ± 0.00	0.82 ± 0.07	1.61 ± 0.16
16/Ca 26	Tholuzham, Thattayil, Adoor, Pathanamthitta	Kerala	15	0.05 ± 0.03	0.04 ± 0.01	0.35 ± 0.02	1.09 ± 0.01
17/Ca 27	Edathitta, Adoor, Pathanamthitta	Kerala	34	ND	0.12 ± 0.03	0.15 ± 0.02	1.04 ± 0.02
18/Ca 29	Kudassanadu, Alappuzha	Kerala	95	ND	0.09 ± 0.01	0.23 ± 0.01	1.48 ± 0.01
19/Ca 30	Kunnamkari, Alappuzha	Kerala	2	ND	0.12 ± 0.01	0.11 ± 0.01	0.78 ± 0.05
20/Ca 31	Muttar, Kaithathod, Alappuzha	Kerala	14	ND	0.16 ± 0.02	0.06 ± 0.00	0.56 ± 0.01
21/Ca 32	Kainery, Alappuzha	Kerala	8	ND	0.10 ± 0.01	0.32 ± 0.02	1.60 ± 0.11
22/Ca 33	Mannanchery, Alappuzha	Kerala	10	0.01 ± 0.00	0.09 ± 0.00	0.25 ± 0.02	1.50 ± 0.03
23/Ca 34	Thalavadi, Alappuzha	Kerala	22	0.02 ± 0.00	0.07 ± 0.01	0.15 ± 0.00	0.92 ± 0.01
24/Ca 35	Poovam, Changanassery, Kottayam	Kerala	20	ND	0.08 ± 0.01	0.22 ± 0.01	1.14 ± 0.01
25/Ca 38	Vaningery, Ancharakotty, Changanassery, Kottayam	Kerala	19	ND	0.07 ± 0.00	0.19 ± 0.01	1.42 ± 0.02
26/Ca 39	Aluva, Ernakulam	Kerala	58	ND	0.05 ± 0.01	0.12 ± 0.04	0.91 ± 0.02
27/Ca 40	Thattekkad, Ernakulam	Kerala	39	ND	0.08 ± 0.01	0.18 ± 0.01	1.10 ± 0.04
28/Ca 41	Bhoothathankett, Ernakulam	Kerala	77	0.01 ± 0.01	0.04 ± 0.00	0.67 ± 0.02	1.30 ± 0.01
29/Ca 42	Wagamon, Idukki	Kerala	1100	ND	0.02 ± 0.01	0.20 ± 0.02	1.18 ± 0.03
30/Ca 43	Nallathanni, Idukki	Kerala	860	0.03 ± 0.01	0.07 ± 0.01	0.11 ± 0.02	0.81 ± 0.03
31/Ca 44	Murinjapuzha, Idukki	Kerala	800	0.01 ± 0.02	0.05 ± 0.02	0.28 ± 0.01	1.75 ± 0.05
32/Ca 45	Pattumala, Peerumade, Idukki	Kerala	1100	ND	0.09 ± 0.03	0.22 ± 0.01	1.54 ± 0.08
33/Ca 47	KFIDC Garden, Elappara, Idukki	Kerala	1180	0.02 ± 0.00	0.13 ± 0.00	2.41 ± 0.06	4.61 ± 0.12
34/Ca 48	Kattappana, Idukki	Kerala	900	0.02 ± 0.00	0.06 ± 0.00	0.71 ± 0.02	5.27 ± 0.13
35/Ca 51	Kumaly, Idukki	Kerala	800	0.06 ± 0.00	0.14 ± 0.02	2.13 ± 0.09	5.10 ± 0.26
36/Ca 52	Kuttikanam, Idukki	Kerala	1064	0.04 ± 0.01	0.12 ± 0.00	1.71 ± 0.09	5.12 ± 0.22
37/Ca 55	Ramakkalmedu, Idukki	Kerala	911	0.27 ± 0.01	0.35 ± 0.00	1.48 ± 0.03	2.56 ± 0.03
38/Ca 56	Periyakanal, Sooryanelli, Idukki	Kerala	1332	0.29 ± 0.01	0.72 ± 0.02	1.23 ± 0.02	4.41 ± 0.06
39/Ca 58	Munnar, Idukki	Kerala	1521	ND	0.07 ± 0.02	0.22 ± 0.05	0.79 ± 0.03
40/Ca 59	Korandikadu, Munnar, Idukki	Kerala	1645	ND	0.20 ± 0.01	0.68 ± 0.02	1.54 ± 0.05
41/Ca 83	Vellanikkara, Thrissur, Kerala	Kerala	30	ND	0.07 ± 0.01	0.15 ± 0.01	0.95 ± 0.01
42/Ca 89	Thenjippalam, Malappuram	Kerala	160	ND	0.04 ± 0.00	0.16 ± 0.02	0.88 ± 0.03
43/Ca 90	Calicut University Campus, Malappuram	Kerala	200	0.02 ± 0.00	0.02 ± 0.00	0.07 ± 0.00	1.65 ± 0.02
44/Ca 92	Kizhakkumpadam, Mukkam, Kozhikode	Kerala	12	0.01 ± 0.00	0.04 ± 0.00	0.55 ± 0.00	0.36 ± 0.01
45/Ca 93	Payyannur, Kannur	Kerala	130	ND	0.03 ± 0.02	0.15 ± 0.00	2.58 ± 0.02
46/Ca 94	Kalpatta, Wayanad	Kerala	760	ND	0.05 ± 0.02	0.15 ± 0.03	1.24 ± 0.18
47/Ca 98	Thirunandikkara, Kanyakumari	Tamil Nadu	110	ND	0.07 ± 0.07	0.11 ± 0.01	0.56 ± 0.01
48/Ca 100	Chittar Dam, Kanyakumari	Tamil Nadu	140	ND	0.08 ± 0.00	0.18 ± 0.01	1.07 ± 0.03
49/Ca 121	Indian Institute of Horticulture Research, Bangalore	Karnataka	883	ND	0.07 ± 0.01	0.07 ± 0.06	0.53 ± 0.01
50/Ca 124	Pilikula, Nisargadhama, Mangalore, Dakshina Kannada	Karnataka	108	ND	0.19 ± 0.06	0.04 ± 0.01	0.15 ± 0.00

ND—not detected; each percentage is an average of six values ± SD.

### Extraction of plant materials

CA plant powder (0.5 g each) was extracted in 50 ml methanol by refluxing for 90 min (16.5 ml × 30 min × 3), extracts were pooled together and concentrated to 5 ml using rotary vacuum evaporator (Buchi, Switzerland) at 40 °C.

### HPTLC-densitometry analysis, calibration of centellosides

Quantitative analyses of four major centellosides (ASA, MDA, ASI, MAD) were carried out by HPTLC-densitometry (CAMAG, Switzerland) made of an automatic Linomat V sample applicator, twin trough plate development chamber, TLC Scanner 3, Reprostar 3 and WinCATS software 4.03. Stock solutions (1 μg/ml) of the standards, MAD, ASI, MDA and ASA, were prepared in methanol; ASI and MAD were applied onto silica gel TLC plates (60 F_254,_ 20 × 10 cm, 0.2 mm thickness) in the range 0.1–0.8 µg/band and 0.2–2.0 µg/band, respectively, and ASA and MDA were spotted in the range 0.05–1.5 µg/band, as 8 mm wide bands (7 tracks), with the Linomat V sample applicator, fitted with a microsyringe under N_2_ flow (application rate 150 nl/s, space between two bands 12.1 mm). (ASA & MDA) and (ASI & MAD) plates were developed up to 80 mm in the twin trough plate development chamber saturated with toluene:ethyl acetate:formic acid (4:5:1, v/v; 20 ml) and organic layer of butanol:ethyl acetate:water (4:1:5, v/v; 20 ml), respectively. Plate(s) were derivatised by spraying with anisaldehyde-sulphuric acid reagent, heated at 110 °C in a hot air oven for 5 min, scanned at 570 nm (tungsten lamp, slit dimension 6.00 × 0.45 mm, scanning speed 20 mm/s) using TLC Scanner 3 and photo documented using Reprostar 3. Calibration plots were generated by plotting against respective peak area(s), and amounts of ASA, MDA, ASI and MAD in CA extracts were determined by means of these calibration plots (Table 2).

**Table 2:** Validation parameters of the four centellosides ASA, MDA, ASI and MAD.

Compound	Regression equation	Correlation coefficient (R^2^)	Linearity range (µg/band)	LOD (µg/band)	LOQ (µg/band)
ASA	y = 4137.1 x + 247.46	0.995	0.05 to 1.25	0.040	0.122
MDA	y = 3662.4 x + 241.83	0.999	0.05 to 1.25	0.022	0.069
ASI	y = 6724.1 x + 465.11	0.998	0.1 to 0.8	0.076	0.230
MAD	y = 2404.8 x + 473.76	0.997	0.2 to 2.0	0.057	0.174

### Quantitative analysis of ASA and MDA

CA extracts (6 µl each) were applied onto silica gel TLC plates (60 F_254,_ 20 × 10 cm, 0.2 mm thickness) as 8 mm wide bands (7 tracks) with the Linomat V sample applicator, fitted with a microsyringe under N_2_ flow (application rate 150 nl/s, space between two bands 12.1 mm). Plates were developed up to 80 mm in the twin trough plate development chamber saturated with 20 ml of toluene:ethyl acetate:formic acid (4:5:1, v/v); peaks of ASA and MDA were well resolved in this mobile phase. Plate(s) were derivatised by spraying with anisaldehyde-sulphuric acid reagent, heated at 110 °C for 5 min, scanned at 570 nm (tungsten lamp, slit dimension 6.00 × 0.45 mm, scanning speed 20 mm/s) using TLC Scanner 3 and photo documented using Reprostar 3.

### Quantitative analysis of ASI and MAD

CA extracts (2 µl each) were applied onto silica gel TLC plates as described in the previous sections (“HPTLC-densitometry analysis, calibration of centellosides” and “Quantitative analysis of ASA and MDA”). Plates were developed up to 80 mm in the twin trough plate development chamber previously saturated with 20 ml organic layer of butanol:ethyl acetate:water (4:1:5, v/v) for 30 min. The twin peaks of ASI and MAD in CA extracts were well resolved in this mobile phase. The plates were derivatised and photo documented.

### Validation methods

HPTLC method was validated in terms of accuracy, precision, repeatability, reproducibility, linearity, limits of detection (LOD), limits of quantification (LOQ) and % recovery^33–35^. Calibration curves were generated by plotting amounts of analytes (standards: ASA, MDA, ASI, MAD) against peak response(s) (Table 2). Intra-day precision was performed by repeating the same assay four times on the same day (of each standard). Inter-day precision was performed by repeating the assay twice for five consecutive days. Recovery of ASA, MDA, ASI and MAD was carried out using standard addition method. Three different concentrations of ASA (0.1, 0.2, 0.3 µg), MDA (0.1, 0.2, 0.3 µg), ASI (0.1, 0.2, 0.3 µg) and MAD (0.25, 0.50, 0.75 µg) were added to pre-analyzed CA extracts and re-analyzed (Table 3). Instrumental precision was determined by applying a sample solution (CA extract, ASA and MDA 6 µl each, ASI and MAD 2 µl each) on a TLC plate, developed as per the protocols described in previous sections, track(s) were scanned repeatedly (ten times each) and % coefficient(s) of variations were determined (Table 4).

**Table 3:** Recovery data of ASA, MDA, ASI and MAD.

Accession no.	ASA + extract (spiked) (µg)	ASA (µg)	ASA in extract (µg)	% Recovery
**ASA**				
CA 56	0.72	0.0	0.72	100
CA 56 + 0.1	0.80	0.1	0.82	97.56
CA 56 + 0.2	0.91	0.2	0.92	98.91
CA 56 + 0.3	1.00	0.3	1.02	98.03

Accession no.	MDA + extract (spiked) (µg)	MDA (µg)	MDA in extract (µg)	% Recovery
**MDA**				
CA 56	0.29	0.0	0.29	100
CA 56 + 0.1	0.38	0.1	0.39	97.44
CA 56 + 0.2	0.48	0.2	0.49	97.96
CA 56 + 0.3	0.57	0.3	0.59	96.61

Accession no.	ASI + extract (spiked) (µg)	ASI (µg)	ASI in extract (µg)	% Recovery
**ASI**				
CA 55	0.74	0.0	0.74	100
CA 55 + 0.1	0.84	0.10	0.84	100
CA 55 + 0.2	0.93	0.20	0.94	98.93
CA 55 + 0.3	1.03	0.30	1.04	99.03

Accession no.	MAD + extract (spiked) (µg)	MAD (µg)	MAD in extract (µg)	% Recovery
**MAD**				
CA 55	1.28	0.0	1.28	100
CA 55 + 0.25	1.51	0.25	1.53	98.69
CA 55 + 0.50	1.76	0.50	1.78	98.88
CA 55 + 0.75	2.03	0.75	2.03	100

**Table 4:** Intra-day and inter-day precision of ASA, MDA, ASI and MAD.

Compound	Concentration (µg/band)	Intra-day precision^a^		Inter-day precision^b^	
		SD in area	% CV	SD in area	% CV
ASA	0.2	31.16	1.99	40.02	2.63
	0.4	40.49	1.79	58.88	2.58
	0.6	20.87	0.70	70.80	2.35
MDA	0.2	15.91	1.62	26.77	2.85
	0.4	32.45	1.86	42.09	2.40
	0.6	48.67	1.81	69.25	2.59
ASI	0.4	40.61	1.35	50.30	1.55
	0.6	70.94	1.74	79.65	1.89
	0.8	76.11	1.49	96.85	1.82
MAD	0.4	34.34	1.83	29.31	1.99
	0.6	37.19	1.42	40.62	1.87
	0.8	39.92	1.24	54.61	1.97

^a^Mean SD of four trials on same day.

^b^Mean SD of two trials for consecutive 5 days; % CV: Coefficient of variation (SD*100/mean).

## Results

### HPTLC-densitometry, validation

**Figure 1:**
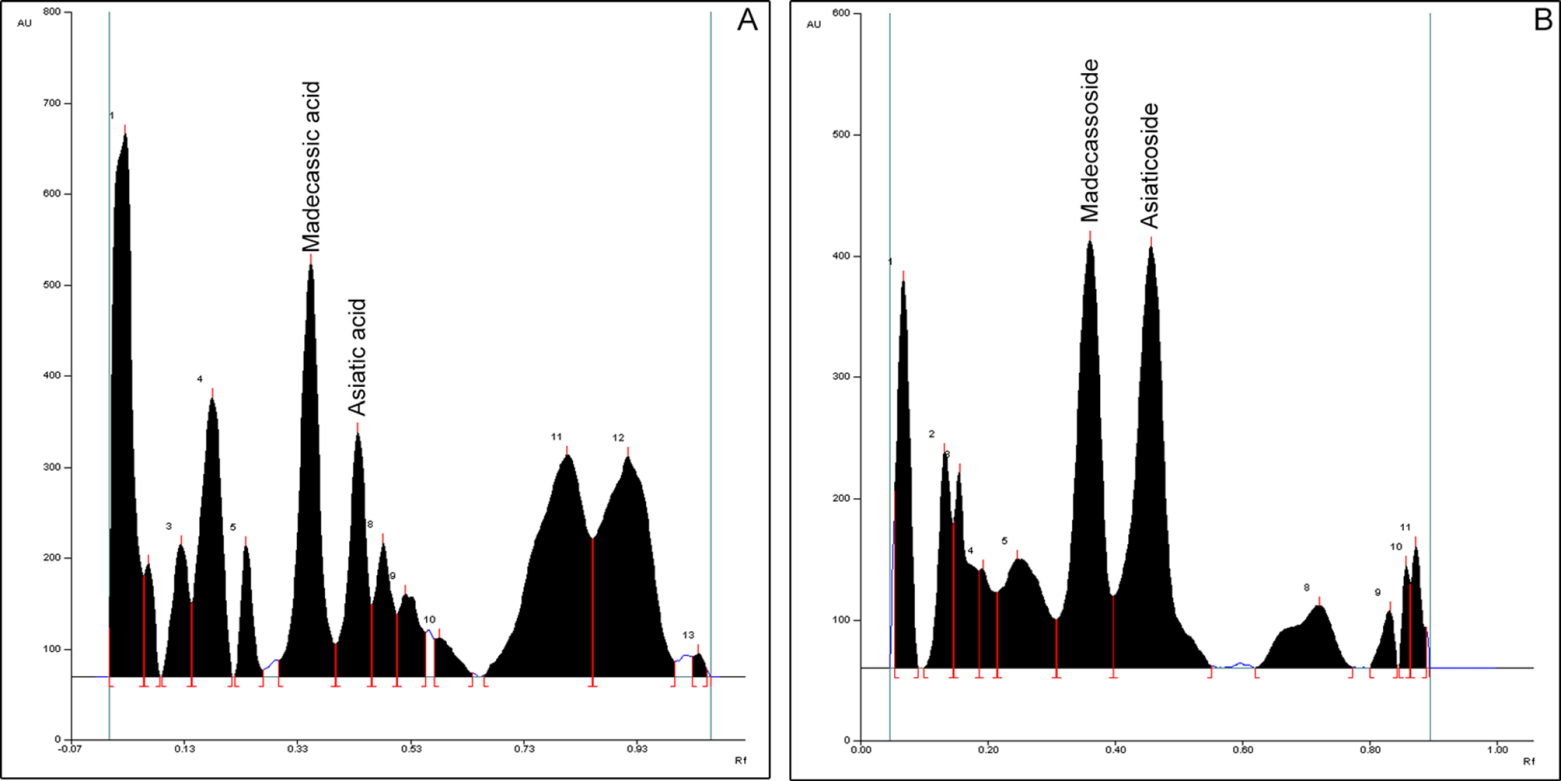
Representative HPTLC chromatograms of methanol extracts of *C. asiatica* accessions, (**A**) Ca-56 (ASA, MDA) and (**B**) Ca-52 (ASI, MAD); data scanning, documentation and analysis by CAMAG TLC Scanner 3, Reprostar 3 and WinCATS software 4.03 respectively.

Fifty CA accessions were collected from the various agro-climatic regions of the south Indian states viz*.*, Kerala, Tamil Nadu and Karnataka, and maintained in a FGB for 3 years. These six generation vegetatively propagated CA specimens grown under identical environmental conditions ensured that the variations in their chemical profiles are genetically determined, and are not influenced by variations in environmental factors (Table 1). Mobile phases for the quantification of aglycones (ASA, MDA) and glycosides (ASI, MAD) were optimized as toluene:ethyl acetate:formic acid (4:5:1, v/v) and organic layer of butanol:ethyl acetate:water (4:1:5, v/v), respectively. R_f_ values of aglycones ASA and MDA were 0.45 ± 0.02 (n = 22) and 0.37 ± 0.02 (n = 50), respectively. Similarly, R_f_ values of ASI and MAD in CA extracts were 0.37 ± 0.02 (n = 50) and 0.47 ± 0.01 (n = 50), respectively. The typical HPTLC chromatograms of CA methanol extracts showed well resolved twin peaks of MDA and ASA (Fig. 1A) and MAD and ASI (Fig. 1B). The quantitative estimation data of the four centellosides are shown in Table 1. HPTLC-densitometry method was validated in terms of linearity, precision, accuracy, reproducibility, LOD, LOQ and % recovery. Reproducibility was assessed by repeated application of standards (ASA, MDA, ASI, MAD); their R_f_ values were reproducible and same as the values observed in CA extracts. Linearity was analyzed using calibration curves of each analyte (ASA: 0.05–1.25 µg/band, MDA: 0.05–1.25 µg/band, ASI: 0.1–0.8 µg/band, MAD: 0.2–2.0 µg/band). Table 2 represents the regression equations, correlation coefficients (R^2^), LODs and LOQs of standards. On repeated measurements 97.56–98.91% (ASA), 96.61–97.44% (MDA), 98.93–100% (ASI) and 98.69–100% (MAD) recoveries were observed for the standards (Table 3). Instrumental precision was determined by scanning the track(s) repeatedly (ten times each) and the % coefficient(s) of variation were acceptable (ASA 1.13, MDA 0.88, ASI 0.81, MAD 0.47) (Table 4).

### Quantification of centellosides

Notably, of the 50 CA accessions, 28 recorded their ASA contents as zero or below the detectable level. Average ASA content in the rest of the 22 CA accessions was 0.06 ± 0.08% (n = 22), with the lowest and highest contents as 0.01% (Ca 13, Ca 15, Ca 22, Ca 33, Ca 41, Ca 44, Ca 92) and 0.29% (CA 56), respectively (Table 1). The lowest MDA content was 0.02% (Ca 42, Ca 90) and highest 0.72% (Ca 56); average 0.12 ± 0.13% (n = 50). MAD content ranged from 0.15% (Ca 124) to 5.27% (Ca 48), and average MAD content was 1.59 ± 1.26% (n = 50). Similarly, the lowest ASI content was displayed by Ca 124 (0.04%) and highest by Ca 47 (2.41%); and average ASI content was 0.44 ± 0.52% (n = 50) (Table 1). The C6-hydroxylation:non-hydroxylation ratios in 50 CA accessions were MDA:ASA 0.12:0.03 (4.00, n = 50, including the 28 accessions with zero ASA content) and MAD:ASI 1.59:0.44 (3.61, n = 50). Likewise, the C28-glycoside:aglycone ratios in these 50 CA accessions viz*.*, ASI:ASA and MAD:MDA were 0.44:0.03 (14.67) and 1.59:0.12 (13.25), respectively (Scheme 1, Table 1).

### Biosynthetic clues

In CA, MDA and MAD are formed by hydroxylation at the C6 position (controlled by CYP450-dependent monooxygenases) of ASA and ASI, respectively. ASI and MAD are formed by C28-glycosylation of ASA and MDA (Scheme 1), respectively, catalyzed by UGTs which link two glucose units and one rhamnose unit to the C28 carboxyl group^3,4,12^_._ MAD also could be formed by C6-hydroxylation and C28-glycosylation of ASA (Scheme 1). In the present study, quantitative estimation of the four major centellosides in 50 CA accessions revealed significant variability in their contents, and the average C28-glycoside:aglycone ratios ASI:ASA and MAD:MDA are 14.67 (n = 50) and 13.25 (n = 50), respectively (Table 1). C6-hydroxylation:non-hydroxylation ratios in 50 CA accessions are MDA:ASA 4.00 (n = 50), MAD:ASI 3.61 (n = 50) (Table 1).

### Elite lines

In this study of 50 south Indian CA accessions ASI, MAD and (ASA + ASI + MDA + MAD) contents ranged from 0.04–2.41%, 0.15–5.27% and 0.38–7.43%, respectively. Industries consider CA accessions (aerial parts) with biomarker (ASI + MAD) content ≥ 4.0% as elite lines, and six accessions are satisfying this benchmark, viz*.*, Ca 51 (7.23%), Ca 47 (7.02%), Ca 52 (6.83%), Ca 48 (5.98%), Ca 56 (5.64%) and Ca 55 (4.04%) (Table 1). Another industrial benchmark (aerial parts) is the biomarker (ASI) content ≥ 2.0%, and two (Ca 47 (2.41%), Ca 51 (2.13%)) out of these 50 accessions are satisfying this criterion. Ca 47 and Ca 51 are fulfilling both the industrial benchmarks for elite lines of CA (Table 1).

## Discussion

The four major centellosides in CA viz*.*, 2 aglycones (ASA, MDA) and 2 glycosides (ASI, MAD), were quantified by various authors using HPLC, HPTLC, LC–MS and other techniques. In an early study, the contents of the 4 major centellosides were quantified by HPLC in five CA (whole plant) samples procured from different locations in Germany (4) and India (1) (all 5 accessions were originally from India)^36^. Glycosides, ASI and MAD contents ranged from (0.18–0.52%) and (0.74–4.02%) and aglycones, ASA and MDA varied from (0.14–0.49%) and (0.53–0.80%), respectively. Average ASI, MAD, ASA and MDA contents were 0.37%, 1.92%, 0.24%, 0.67%. Overall, C6-hydroxylation:non-hydroxylation (MDA:ASA and MAD:ASI) ratios were 0.67:0.24 (2.79) and 1.92:0.37 (5.19) and C28-glycoside:aglycone ratios ASI:ASA and MAD:MDA were 1.54 and 2.87. The total centelloside contents ranged from 1.98–5.26%^36^. Again, Schaneberg and co-workers analyzed ASI, MAD, ASA and MDA in extracts of three CA samples obtained from Boulder (USA), Norway, (USA) and Sri Lanka by HPLC. The average contents of ASI, MAD, ASA and MDA were 0.64%, 0.88%, 0.13% and 0.28%, respectively (MDA:ASA 2.15; MAD:ASI 1.38 & ASI:ASA 4.92; MAD:MDA 3.14)^25^. Randriamampionona and co-workers quantified the four centellosides in seven CA leaf samples collected from various habitats in Madagascar by HPLC, and the average ASI, MAD, ASA and MDA contents were 3.85%, 3.63%, 0.30% and 0.28% (dry wt.) (MDA:ASA 0.93; MAD:ASI 0.94 & ASI:ASA 12.83; MAD:MDA 12.96)^26^. Rafamantanana et al. analyzed three CA leaf samples collected from the East and High Plateau regions of Madagascar using HPLC. Average ASI, MAD, ASA and MDA contents were 1.79%, 1.54%, 0.57% and 0.56% (dry wt.) (MDA:ASA 0.98; MAD:ASI 0.86 & ASI:ASA 3.14; MAD:MDA 2.75). One CA sample showed below detectable (zero) levels of ASA and MDA^21^. Again, ASI (0.21%), MAD (0.18%), ASA (0.46%) and MDA (0.31%) (n = 1, dry wt.) contents in CA leaves obtained from Bad Grund, Germany were quantified by Raj and Kielisz (2019) by HPTLC-densitometry (MDA:ASA 0.67; MAD:ASI 0.86 & ASI:ASA 0.46; MAD:MDA 0.58)^37^.

Randriamampionona and co-workers also reported the average ASI, MAD, ASA and MDA contents in in vitro propagated CA leaves as 1.06%, 0.84%, 0.10% and 0.14% (n = 7) (dry wt.) (MDA:ASA 0.71; MAD:ASI 0.79 & ASI:ASA 7.57; MAD:MDA 8.40)^26^. James and co-workers determined the average ASI, MAD, ASA and MDA contents in two in vitro propagated CA leaf samples by HPLC as 4.88%, 4.52%, 1.84%, 1.93% (dry wt.) (MDA:ASA 1.05; MAD:ASI 0.93 & ASI:ASA 2.65; MAD:MDA 2.34)^20^. Prasad and co-workers quantified the average ASI, MAD, ASA and MDA contents in hydroponically grown (42nd day of growth) CA whole plants by HPLC as 0.17%, 1.10%, 0.63%, 3.66% (dry wt.) (MDA:ASA 5.81; MAD:ASI 6.47 & ASI:ASA 0.27; MAD:MDA 0.30). In these hydroponically grown plants the glycoside contents are low compared to the aglycones^38^. Fourteen CA (leaves) accessions grown under identical glass house conditions minimizing the effect of environmental parameters were analyzed for their centellosides by HPLC and found average ASI, MAD, ASA and MDA contents as 0.13%, 0.08%, 0.05%, 0.13% (n = 14, fresh wt.) (MDA:ASA 2.60; MAD:ASI 0.62 & ASI:ASA 2.60; MAD:MDA 0.62)^31^. In another study, Prasad and co-workers estimated the average ASI, MAD, ASA and MDA contents in three sets of in vitro cultures (leaf tissues) developed on the basis of an artificial neural network-based model by HPLC as 0.12%, 0.05%, 0.86%, 2.03% (dry wt.) (MDA:ASA 2.36; MAD:ASI 0.42 & ASI:ASA 0.14; MAD:MDA 0.02)^39^.

In these quantification studies, the number of CA samples is limited, and they were originally from markedly varying environmental (natural habitats)^21,25,26,36,37^ and growth (in vitro)^20,26,31,38,39^ conditions. Along with the genetic origins, the variation in collection (ecological)/growth conditions influence the C6-hydroxylation (MDA:ASA; MAD:ASI) and C28-glycosylation (ASI:ASA; MAD:MDA) reactions^4,40^.

In previous studies, the C6-hydroxylation:non-hydroxylation ratios in CA accessions from natural habitats were (MDA:ASA 2.79; MAD:ASI 5.19)^36^, (MDA:ASA 2.15; MAD:ASI 1.38)^25^, (MDA:ASA 0.93; MAD:ASI 0.94)^26^, (MDA:ASA 0.98; MAD:ASI 0.86)^21^, (MDA:ASA 0.67; MAD:ASI 0.86)^37^. Similarly, C6-hydroxylation:non-hydroxylation ratios in in vitro or controlled conditions were (MDA:ASA 0.71; MAD:ASI 0.79)^26^, (MDA:ASA 1.05; MAD:ASI 0.93)^20^, (MDA:ASA 5.81; MAD:ASI 6.47)^38^, (MDA:ASA 2.60; MAD:ASI 0.62)^31^, (MDA:ASA 2.36; MAD:ASI 0.42)^39^.

In most previous studies, glycosides are dominant in CA tissues compared to aglycones. Glycoside:aglycone ratios (in CA accessions from natural habitats) were (ASI:ASA 1.54, MAD:MDA 2.87)^36^, (ASI:ASA 4.92, MAD:MDA 3.14)^25^, (ASI:ASA 12.83; MAD:MDA 12.96)^26^, (ASI:ASA 3.14; MAD:MDA 2.75)^21^. Glycoside:aglycone ratios in in vitro or controlled conditions were (ASI:ASA 7.57; MAD:MDA 8.40)^26^, (ASI:ASA 2.65; MAD:MDA 2.34)^20^. In a few cases, aglycone contents were relatively high viz*.*, glycoside:aglycone ratios (CA accessions from natural habitats): (ASI:ASA 0.46; MAD:MDA 0.58)^37^; glycoside:aglycone ratios (in vitro or controlled conditions): (ASI:ASA 0.27; MAD:MDA 0.30)^38^, (ASI:ASA 2.60; MAD:MDA 0.62)^31^, (ASI:ASA 0.14; MAD:MDA 0.02)^39^. In fact, in these studies no consistent patterns were observed in C6-hydroxylation:non-hydroxylation and C28-glycoside:aglycone ratios in CA samples both from natural habitats or grown in vitro; moreover the sample sizes are limited, and their growing conditions, plant part, sample preparation, extraction and analytical parameters varied substantially.

In the present study, 50 CA accessions collected from various agroclimatic conditions were grown under identical ecological conditions and their C6-hydroxylation:non-hydroxylation (MDA:ASA; MAD:ASI) and C28-glycoside:aglycone (ASI:ASA; MAD:MDA) ratios were analyzed under standardized (identical) parameters. Therefore, these ratios viz*.*, MDA:ASA (4.00, n = 50, including the 28 accessions with zero ASA content), MAD:ASI (3.61, n = 50), ASI:ASA (14.67, n = 50), MAD:MDA (13.25, n = 50), are genetically determined (controlled), and reliable bioconversion and pharmaceutical clues can be derived from these data. Thereby, our study indicates that the C6-hydroxylation and C28-glycosylation driven by CYP450-dependent monooxygenases and UGTs, respectively, are leading bioconversion steps in CA.

In all 50 CA accessions, except in Ca 92 (ASI 0.55%, MAD 0.36%), MAD contents are higher than ASI (including the ASI-rich accessions: Ca 47, Ca 51, Ca 52, Ca 55, Ca 56). Similarly, in our previous study over a decade ago^28^, 60 CA accessions were originally collected from a wide range of locations in south India and the Andaman Islands (as in the present study) and grown under identical ecological conditions for 3 generations. ASI and MAD contents of these 60 CA accessions were quantified by similar HPTLC-densitometry protocol, and the average MAD:ASI ratio was 1.86:0.37 (i.e., 3.96, n = 60, which is close to our current ratio of 3.61). Only one of these accessions showed higher ASI content (0.80%) compared to MAD (0.29%). Of these 60 CA accessions, one showed absence of both the glycosides (ASI, MAD) and two other accessions showed the absence of ASI^28^. In another recent study, we screened 106 CA accessions collected from various natural habitats in south India (i.e., directly from different ecological conditions) using similar protocol, and the observed MAD:ASI average ratio was 1.22:0.55 = 2.71 (unpublished data). This ratio (2.71) is considerably different from the two screening studies viz*.*, current study MAD:ASI 3.61, Thomas and co-workers MAD:ASI 3.96^28^, under identical ecological conditions. In these 106 CA accessions, 6 showed higher ASI contents compared to MAD levels (unpublished data). These extensive data (under identical and varying ecological conditions) demonstrate MAD as the prominent constituent of the four centellosides in CA. These results clearly support the bioconversion possibilities portrayed in Scheme 1.

On further evaluation of the data, among the four major centellosides in CA, ASA which is formed by terpenoid biosynthesis has three bioconversion probabilities, viz*.*, ASA to MDA (C6-hydroxylation), ASA to ASI (C28-glycosylation) and ASA to MAD (C6-hydroxylation + C28-glycosylation) (Scheme 1), whereas MDA and ASI have only one (each) possibility of getting converted to MAD by C28-glycosylation and C6-hydroxylation, respectively. In 50 CA accessions under identical environmental conditions, the average contents of ASA, MDA, ASI and MAD are 0.03, 0.12, 0.44 and 1.59%, respectively. As anticipated, ASA (aglycone) which has highest probability of enzymatic conversion(s), showed the lowest content in CA. MDA (aglycone) % content is 4 times that of ASA, and the highest content was displayed by MAD (1.59%) which has no further conversion prospects (Scheme 1). The average content of MAD (1.59%, n = 50) is 2.69 times the total average contents of the other three centellosides (ASA + MDA + ASI = 0.59%, n = 50). These data are inferring high rates (probabilities) of one or more transformations of the three centellosides as depicted in Scheme 1. The enzymes involved in C6-hydroxylation^13,16^ and C-28 glycosylation^3,4,12^ reactions in CA are already elucidated by biosynthetic studies.

ASI and (ASI + MAD) have been widely assigned as the major biomarkers for quality evaluation of CA raw drugs, pharmaceutical and cosmetic products^23,28^. But the four centellosides, ASA, MDA ASI and MAD, exert varying effects in the biological (neuroprotection, wound healing, skin protection) activities of CA, and thereby display disproportionate influences (roles) in its pharmaceutical applications. In our study, CA accessions with their ecological variations nullified, showed considerable fluctuations (even absence) in their ASA, ASI, MDA and MAD contents. Therefore, efficient quality control practices of CA raw drugs warrant the quantification of a set of major triterpenoids (four centellosides) as biomarkers^23^.

Apart from deductions on centelloside bioconversions, this study discovered six elite clones of CA from south India with their (ASI + MAD) contents above the industrial benchmark (≥ 4%). Two elite clones with ASI contents ≥ 2% were also identified. The agricultural practices of these CA elite lines can be standardized, and they can utilized for industrial purposes. All six elite accessions of CA discovered in this study are from high altitude locations (800–1332 m MSL) in the Idukki district in the south Indian state of Kerala. In our previous search (screening) for CA elite lines over a decade ago, two of the highest bioactive (ASI + MAD) yielding accessions were from high altitudes in Idukki district^28^. Our studies discovered a hotspot of high-yielding CA accessions in south India.

## Conclusions

Briefly, in an ecologically uniform environment, 50 CA accessions showed the (ASI:ASA) and (MAD:MDA) C28-glycosylation ratios as 14.67 and 13.25, respectively, displaying relatively high contents of glycosides (MAD, ASI) in them. Similarly, the C6 hydroxylation (MDA:ASA and MAD:ASI) ratios in 50 CA accessions were 4.00 (n = 50) and 3.61 (n = 50). Our study, infers that the two defined triterpenoid skeletal modifications at C6 (hydroxylation) and C28 (glycosylation) are dominant bioconversion steps in CA.

The high demand of CA is leading to its overexploitation at an uncontrolled rate and destruction of its wild genotypes^1^. Our study discovered six elite lines of CA from south India; the agro-practices of these high yielding genotypes can be standardized and utilized for its pharmaceutical and cosmetic purposes. The four major centellosides viz*.*, ASA, ASI, MDA and MAD, have uneven effects on the bioactivities of CA based extracts/drugs. In this study, ASA was below detectable levels in 28 of the 50 screened CA accessions. These facts emphasize the need for quantifying the contents of all the four centellosides (as biomarkers) in CA extracts, pharmaceutical and cosmetic products.

## Data Availability

All data generated or analyzed during this study are included in this article.

## Abbreviations

ASA: Asiatic acid
ASI: Asiaticoside
CA: *Centella asiatica*
CYP: Cytochrome P450
FGB: Field Gene Bank
MDA: Madecassic acid
MAD: Madecassoside
Glu: Glucose
HPLC: High Performance Liquid Chromatography
HPTLC: High Performance Thin Layer Chromatography
LC–MS: Liquid Chromatography–Mass Spectrometry
Rha: Rhamnose
UGT: UDP-Glc glucosyltransferase
LOD: Limit of detection
LOQ: Limits of quantification
